# Emergency department involvement in the diagnosis of cancer among older adults: a SEER-Medicare study

**DOI:** 10.1093/jncics/pkae039

**Published:** 2024-05-25

**Authors:** Caroline A Thompson, Paige Sheridan, Eman Metwally, Sharon Peacock Hinton, Megan A Mullins, Ellis C Dillon, Matthew Thompson, Nicholas Pettit, Allison W Kurian, Sandi L Pruitt, Georgios Lyratzopoulos

**Affiliations:** Department of Epidemiology, Gillings School of Global Public Health, University of North Carolina, Chapel Hill, NC, USA; Lineberger Comprehensive Cancer Center, University of North Carolina, Chapel Hill, NC, USA; Aetion, Inc, New York, NY, USA; Department of Epidemiology, Gillings School of Global Public Health, University of North Carolina, Chapel Hill, NC, USA; Department of Epidemiology, Gillings School of Global Public Health, University of North Carolina, Chapel Hill, NC, USA; Peter O’Donnell Jr School of Public Health, UT Southwestern Medical Center, Dallas, TX, USA; Simmons Comprehensive Cancer Center, UT Southwestern Medical Center, Dallas, TX, USA; Center on Aging, UConn Health, Farmington, CT, USA; Department of Family Medicine, University of Washington, Seattle, WA, USA; Department of Emergency Medicine, Indiana University School of Medicine, Indianapolis, IN, USA; Stanford University School of Medicine, Stanford, CA, USA; Peter O’Donnell Jr School of Public Health, UT Southwestern Medical Center, Dallas, TX, USA; Simmons Comprehensive Cancer Center, UT Southwestern Medical Center, Dallas, TX, USA; Epidemiology of Cancer Healthcare & Outcomes, Institute of Epidemiology & Health Care, University College London, London, UK

## Abstract

**Background:**

Internationally, 20% to 50% of cancer is diagnosed through emergency presentation, which is associated with lower survival, poor patient experience, and socioeconomic disparities, but population-based evidence about emergency diagnosis in the United States is limited. We estimated emergency department (ED) involvement in the diagnosis of cancer in a nationally representative population of older US adults, and its association with sociodemographic, clinical, and tumor characteristics.

**Methods:**

We analyzed Surveillance, Epidemiology, and End Results Program–Medicare data for Medicare beneficiaries (≥66 years old) with a diagnosis of female breast, colorectal, lung, and prostate cancers (2008-2017), defining their earliest cancer-related claim as their index date, and patients who visited the ED 0 to 30 days before their index date to have “ED involvement” in their diagnosis, with stratification as 0 to 7 or 8 to 30 days. We estimated covariate-adjusted associations of patient age, sex, race and ethnicity, marital status, comorbidity score, tumor stage, year of diagnosis, rurality, and census-tract poverty with ED involvement using modified Poisson regression.

**Results:**

Among 614 748 patients, 23% had ED involvement, with 18% visiting the ED in the 0 to 7 days before their index date. This rate varied greatly by tumor site, with breast cancer at 8%, colorectal cancer at 39%, lung cancer at 40%, and prostate cancer at 7%. In adjusted models, older age, female sex, non-Hispanic Black and Native Hawaiian or Other Pacific Islander race, being unmarried, recent year of diagnosis, later-stage disease, comorbidities, and poverty were associated with ED involvement.

**Conclusions:**

The ED may be involved in the initial identification of cancer for 1 in 5 patients. Earlier, system-level identification of cancer in non-ED settings should be prioritized, especially among underserved populations.

Cancer diagnosis in the emergency department (ED) as opposed to another ambulatory setting is a widely studied problem outside the United States. It is estimated that 20% to 50% of patients with cancer worldwide receive their initial diagnosis via an “emergency presentation” health-care system pathway. A growing body of international literature has demonstrated that emergency presentation is associated with lower overall survival (1-3), poorer patient experience (4), and sociodemographic disparities (1,3,5). Research from US populations suggest that 5% to 40% of patients with cancer in the United States may be emergency presentations, depending on the tumor site and population (6-12); in addition, it is more common for patients who are older, Black, unmarried, and in a lower income bracket and who have more comorbidities and no usual source of care (6-9). The scope of this evidence is limited, however, and primarily derived from hospital-based studies. The definition of *emergency diagnosis* has not been consistent, and the most recent population-based data (for 1 cancer site) is now 20 years old (7). In this study, we estimated how often ED utilization occurred at the start of the diagnostic episode (as “ED involvement”) for Medicare-enrolled patients with breast, colorectal, lung, or prostate cancers over a recent 10-year period and assessed the importance of associated patient demographic and clinical factors. To our knowledge, this is the first study of emergency diagnosis of multiple cancer types in a nationally representative US population.

## Methods

### Study data and population

We analyzed the Surveillance, Epidemiology, and End Results (SEER) Program–Medicare database, which is a linkage of 2 large population-based data sources that capture detailed information about Medicare beneficiaries with cancer (13). Medicare is a US federal health insurance program; most adults aged 65 years and older as well as younger persons with disabilities or end-stage renal disease are eligible to enroll. Medicare data include information about all aspects of covered health-care services, from the start of eligibility until death. Cancer details are derived from the SEER Program, a consortium of 22 population-based regional and statewide cancer registries, which collects patient demographics, tumor characteristics, initial treatment, and outcomes for all cancer diagnoses. Ninety-four percent of US older adults are Medicare beneficiaries, and the SEER Program covers 48% of the US population, but SEER-Medicare does not have claims for beneficiaries enrolled in Medicare managed care plans; this proportion was 10% in 2008 and grew to 20% in 2017 (14-17).

Our study population included Medicare beneficiaries aged 66 years or older who had a diagnosis of colorectal, lung, female breast, or prostate cancer between 2008 and 2017. We excluded beneficiaries who were younger (<66 years old), had prior cancer, were diagnosed after death, or had in situ or nonmalignant disease. To ensure valid estimation of the Charlson Comorbidity Index score and health-care utilization in the year before diagnosis, we also excluded anyone not continuously enrolled in Medicare fee for service or who had evidence of managed care plan enrollment in the year before diagnosis. This study was reviewed as exempt by The University of North Carolina Institutional Review Board (No. 22-2998).

### Index date

To characterize the health-care episode in which the cancer was first detected, we used a published and validated algorithm (18) that identifies the earliest *International Statistical Classification of Diseases, Tenth Revision or International Classification of Diseases, Ninth Revision* claim code associated with the patient’s tumor site within ±1 month of the SEER diagnosis month. This index date approximates the start of the cancer care episode (ie, when care delivery for suspected cancer began). Using this method, we successfully assigned index dates for 86.9% of study participants. For the remaining 13.1% of patients, the index date was imputed as the 15th of the SEER diagnosis month (Table 1).

**Table 1.** Characteristics of SEER-Medicare patients diagnosed with breast, colorectal, lung, or prostate cancer (2008-2017)^a^

Characteristic	All	Imputed or exact index date		Primary cancer site			
		Exact	Imputed	Breast	Colorectal	Lung	Prostate
All, No. (%)	614 748 (100)	534 351 (86.9)	80 397 (13.1)	139 068 (22.6)	111 955 (18.2)	186 348 (30.3)	177 377 (28.9)
Sex, No. (%)							
Male	320 512 (52.1)	283 663 (53.1)	36 849 (45.8)	NA	51 486 (46.0)	91 649 (49.2)	177 377 (100)
Female	294 236 (47.9)	250 688 (46.9)	43 548 (54.2)	139 068 (100)	60 469 (54.0)	94 699 (50.8)	NA
Age, mean (SD), y	75.9 (7.2)	75.8 (7.1)	76.4 (7.6)	75.9 (7.3)	78.2 (7.7)	76.4 (7.0)	73.9 (6.3)
66-69 y, No. (%)	138 295 (22.5)	120 334 (22.5)	17 961 (22.3)	32 184 (23.1)	17 912 (16.0)	35 991 (19.3)	52 208 (29.4)
70-74 y, No. (%)	163 396 (26.6)	143 819 (26.9)	19 577 (24.4)	36 240 (26.1)	23 208 (20.7)	47 330 (25.4)	56 618 (31.9)
75-79 y, No. (%)	130 936 (21.3)	114 521 (21.4)	16 415 (20.4)	28 673 (20.6)	23 184 (20.7)	42 653 (22.9)	36 426 (20.5)
80-84 y, No. (%)	95 362 (15.5)	82 413 (15.4)	12 949 (16.1)	21 473 (15.4)	21 569 (19.3)	33 327 (17.9)	18 993 (10.7)
≥85 y, No. (%)	86 759 (14.1)	73 264 (13.7)	13 495 (16.8)	20 498 (14.7)	26 082 (23.3)	27 047 (14.5)	13 132 (7.4)
Race and ethnicity, No. (%)							
American Indian or Alaska Native	2111 (0.3)	1750 (0.3)	361 (0.4)	440 (0.3)	460 (0.4)	626 (0.3)	585 (0.3)
Asian American	23 138 (3.8)	19 976 (3.7)	3162 (3.9)	4871 (3.5)	5354 (4.8)	7213 (3.9)	5700 (3.2)
Hispanic	32 080 (5.2)	27 935 (5.2)	4145 (5.2)	7016 (5.0)	6562 (5.9)	7440 (4.0)	11 062 (6.2)
Native Hawaiian or Other Pacific Islander	1533 (0.2)	1279 (0.2)	254 (0.3)	376 (0.3)	230 (0.2)	475 (0.3)	452 (0.3)
Non-Hispanic Black	51 251 (8.3)	43 491 (8.1)	7760 (9.7)	10 267 (7.4)	9124 (8.1)	13 448 (7.2)	18 412 (10.4)
Non-Hispanic White	498 859 (81.1)	434 538 (81.3)	64 321 (80.0)	115 428 (83.0)	89 767 (80.2)	156 834 (84.2)	136 830 (77.1)
Mixed race	298 (0.0)	252 (0.0)	46 (0.1)	72 (0.1)	53 (0.0)	85 (0.0)	88 (0.0)
Other or unknown race	5478 (0.9)	5130 (1.0)	348 (0.4)	598 (0.4)	405 (0.4)	227 (0.1)	4248 (2.4)
Married or domestic partner, No. (%)							
No	184 182 (30.0)	155 489 (29.1)	28 693 (35.7)	51 271 (36.9)	39 286 (35.1)	65 963 (35.4)	27 662 (15.6)
Yes	230 216 (37.4)	203 151 (38.0)	27 065 (33.7)	44 576 (32.1)	37 735 (33.7)	64 552 (34.6)	83 353 (47.0)
Unknown	200 350 (32.6)	175 711 (32.9)	24 639 (30.6)	43 221 (31.1)	34 934 (31.2)	55 833 (30.0)	66 362 (37.4)
Year of diagnosis, No. (%)							
2008	71 177(11.6)	61 946 (11.6)	9231 (11.5)	14 282 (10.3)	13 884 (12.4)	20 884 (11.2)	22 127 (12.5)
2009	68 058 (11.1)	59 265 (11.1)	8793 (10.9)	14 152 (10.2)	12 975 (11.6)	20 418 (11.0)	20 513 (11.6)
2010	64 742 (10.5)	56 282 (10.5)	8460 (10.5)	13 613 (9.8)	12 205 (10.9)	19 439 (10.4)	19 485 (11.0)
2011	64 055 (10.4)	56 111 (10.5)	7944 (9.9)	13 778 (9.9)	11 781 (10.5)	18 821 (10.1)	19 675 (11.1)
2012	59 817 (9.7)	51 972 (9.7)	7845 (9.8)	13 832 (9.9)	11 205 (10.0)	18 814 (10.1)	15 966 (9.0)
2013	57 970 (9.4)	50 291 (9.4)	7679 (9.6)	13 788 (9.9)	10 553 (9.4)	18 174 (9.8)	15 455 (8.7)
2014	56 757 (9.2)	49 118 (9.2)	7639 (9.5)	13 828 (9.9)	10 283 (9.2)	17 879 (9.6)	14 767 (8.3)
2015	57 449 (9.3)	49 780 (9.3)	7669 (9.5)	13 961 (10.0)	10 146 (9.1)	17 747 (9.5)	15 595 (8.8)
2016	58 056 (9.4)	50 354 (9.4)	7702 (9.6)	14 164 (10.2)	9 654 (8.6)	17 422 (9.3)	16 816 (9.5)
2017	56 667 (9.2)	49 232 (9.2)	7435 (9.2)	13 670 (9.8)	9 269 (8.3)	16 750 (9.0)	16 978 (9.6)
SEER tumor stage, No. (%)							
Localized	307 060 (49.9)	275 409 (51.5)	31 651 (39.4)	94 549 (68.0)	42 396 (37.9)	37 620 (20.2)	132 495 (74.7)
Regional metastasis	130 420 (21.2)	114 905 (21.5)	15 515 (19.3)	32 049 (23.0)	40 016 (35.7)	41 165 (22.1)	17 190 (9.7)
Distant metastasis	142 167 (23.1)	117 915 (22.1)	24 252 (30.2)	8 858 (6.4)	22 385 (20.0)	97 308 (52.2)	13 616 (7.7)
Unknown	35 101 (5.7)	26 122 (4.9)	8979 (11.2)	3612 (2.6)	7158 (6.4)	10 255 (5.5)	14 076 (7.9)
Charlson Comorbidity Index score, No. (%)							
0	325 185 (52.9)	281 846 (52.7)	43 339 (53.9)	81 237 (58.4)	57 100 (51.0)	77 338 (41.5)	109 510 (61.7)
1	141 682 (23.0)	124 365 (23.3)	17 317 (21.5)	31 105 (22.4)	25 362 (22.7)	48 531 (26.0)	36 684 (20.7)
2	70 659 (11.5)	61 563 (11.5)	9096 (11.3)	13 808 (9.9)	13 330 (11.9)	27 356 (14.7)	16 165 (9.1)
≥3	77 222 (12.6)	66 577 (12.5)	10 645 (13.2)	12 918 (9.3)	16 163 (14.4)	33 123 (17.8)	15 018 (8.5)
County geography, No. (%)							
Metropolitan	520 920 (84.7)	454 068 (85.0)	>66 845 (>83.2)	119 910 (86.2)	>94 396 (>84.3)	>155 310 (>83.3)	151 298 (85.3)
Urban, nonmetropolitan	83 507 (13.6)	71 470 (13.4)	12 037 (15.0)	17 196 (12.4)	15 577 (13.9)	27 455 (14.7)	23 279 (13.1)
Rural	10 245 (1.7)	8741 (1.6)	1504 (1.9)	1936 (1.4)	1971 (1.8)	3572 (1.9)	2 766 (1.6)
Unknown	76 (0.0)	>65 (0.0)	<11 (0.0)	26 (0.0)	<11 (0.0)	<11 (0.0)	34 (0.0)
Census-tract poverty indicator, No. (%)							
<5% poverty	138 041 (22.5)	122 030 (22.8)	16 011 (19.9)	33 682 (24.2)	23 694 (21.2)	37 137 (19.9)	43 528 (24.5)
5%-10% poverty	156 277 (25.4)	136 882 (25.6)	19 395 (24.1)	36 881 (26.5)	27 845 (24.9)	45 359 (24.3)	46 192 (26.0)
10%-20% poverty	165 348 (26.9)	142 656 (26.7)	22 692 (28.2)	36 500 (26.2)	30 768 (27.5)	51 168 (27.5)	46 912 (26.4)
>20% poverty	106 775 (17.4)	91 626 (17.1)	15 149 (18.8)	22 004 (15.8)	21 048 (18.8)	34 424 (18.5)	29 299 (16.5)
Census tract unknown	48 307 (7.9)	41 157 (7.7)	7150 (8.9)	10 001 (7.2)	8600 (7.7)	18 260 (9.8)	11 446 (6.5)
SEER registry region							
East	243 682 (39.6)	213 114 (39.9)	30 568 (38.0)	56 203 (40.4)	46 167 (41.2)	74 186 (39.8)	67 126 (37.8)
South	121 633 (19.8)	105 183 (19.7)	16 450 (20.5)	25 203 (18.1)	21 234 (19.0)	41 152 (22.1)	34 044 (19.2)
Midwest	53 913 (8.8)	47 032 (8.8)	6881 (8.6)	11 652 (8.4)	10 408 (9.3)	16 867 (9.1)	14 986 (8.4)
West	195 520 (31.8)	169 022 (31.6)	26 498 (33.0)	46 010 (33.1)	34 146 (30.5)	54 143 (29.1)	61 221 (34.5)

a NA = not available; SEER = Surveillance, Epidemiology, and End Results.

### Outcome

Following precedent (3), our primary (binary) outcome was whether each patient visited the ED in the 30 days before their index date, which we considered “ED involvement” in their initial cancer diagnosis. First, a 1-month lookback period starting from the index date (day 0) to 1 month (day ‒30) before was established. Next, ED utilization during the lookback period was defined as either any inpatient hospitalization with an associated ED claim or any outpatient claims with an ED revenue code (0450-0459). To explore the timing of ED utilization, this interval was subclassified as 0 to 7 (including ED visits on the index date) or 8 to 30 days before the index date. For patients whose index date occurred during an emergency (nonelective) in-patient hospitalization, this interval was set to 0.

### Exposure variables

We classified patient demographic, socioeconomic, and clinical characteristics using SEER data: age at diagnosis, sex, race and ethnicity (Non-Hispanic White, Non-Hispanic Black, Hispanic, Asian American [including individuals of Chinese, Japanese, Filipino, Korean, Vietnamese, Asian Indian, or Other Asian ethnicity], American Indian or Alaska Native, Native Hawaiian or Pacific Islander, mixed race, other or unknown race), partnership status at diagnosis (married or partnered, not married/partnered, unknown), year of diagnosis, SEER region (East, Midwest, South, or Western United States), urban-rural residence (metropolitan counties, urban nonmetropolitan counties, rural counties), and census-tract level poverty (<5%, 5%-10%, >10%-20%, or >20%) in the year of diagnosis. Summary tumor stage (localized, regional, distant) is generally captured after initial presentation but was included as a proxy for the extent of disease at presentation. A claims-based modified Charlson Comorbidity Index score was calculated based on all claims in the year before diagnosis (19,20).

### Statistical analyses

All analyses were performed using SAS, version 9.4, statistical software (SAS Institute Inc, Cary, NC). Descriptive statistics were calculated overall, by imputed vs exact index date, and by ED visit timing before the index date (0-7 days or 8-30 days). We suppressed cell counts below 11 and rounded proportions to whole numbers for in-text callouts. With ED involvement as the outcome, we used modified Poisson regression with robust variance estimation to estimate prevalence ratios and corresponding 95% confidence intervals (CIs) using the SAS *PROC GENMOD* statement (21,22). A random intercept was included for clustering by registry, but the intraclass correlation coefficient was low (<1%); therefore, it was treated as a fixed effect in all models. All analyses were conducted overall and by cancer site, excluding patients with unknown values of covariates, except for unknown partner status, which we treated as a separate category. Variables included in all models were age at diagnosis; year of diagnosis; tumor stage; SEER region and registry; imputed vs exact index date; number of outpatient-, inpatient-, and ED-visit-days in the year before diagnosis; and tumor site for “all cancers” models. Covariate adjustment sets for each additional exposure of interest were defined using directed acyclic graphs (23) (Supplementary Methods, available online).

### Sensitivity analyses

Because 13% of our population had their index date imputed as the 15th of the SEER diagnosis month due to lack of cancer-related claims, we excluded them from all main analyses.

## Results

The starting population size was 2 110 0096 SEER region residents of any age. After applying all exclusion criteria, including restricting to those individuals 66 years of age and older with continuous Medicare fee-for-service coverage, the final study population was 614 748 people (Supplementary Figure 1, available online). The average age at diagnosis was 76 years, half the population was male, 81% were non-Hispanic White, 8% were non-Hispanic Black, 5% were Hispanic, 4% were Asian American, fewer than 1% were Native Hawaiian or Other Pacific Islander or American Indian or Alaska Native. Marital status at diagnosis was distributed evenly, with 33% of each: married or partnered, unmarried, unknown. About half had a Charlson Comorbidity Index score of 0. Patients without an exact index date were slightly older and more frequently female, diagnosed with later-stage cancer, and residing in high-poverty regions (Table 1).

**Figure 1.**
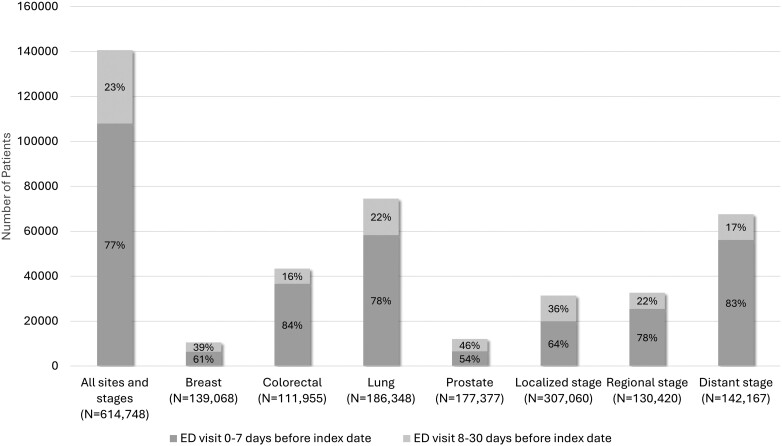
Number of patients with ED involvement in their diagnosis among Surveillance, Epidemiology, and End Results Program–Medicare patients diagnosed with breast, colorectal, lung, or prostate cancers (2008-2017), by timing of the ED visit relative to the index date (0-7 days vs 8-30 days before the index date). Figures shown for all patients combined and by tumor site (breast, colorectal, lung, prostate) and disease stage at diagnosis (localized, regional metastasis, or distant metastasis). ED = emergency department.

Across all cancer sites, 23% of patients had ED involvement, with marked variation by tumor site: lung cancer at 40%, colorectal cancer (CRC) at 39%, breast cancer at 8%, and prostate cancer at 7% (Figure 1). Older patients (30% aged >85 years vs 16% aged 66-69 years) and unpartnered patients (30% vs 18% in patients with spouses or partners) had higher ED involvement. ED involvement increased from 22% in 2008 to 24% in 2014, then decreased to 23% in 2017. Non-Hispanic Black patients (28%) as well as American Indian or Alaska Native and Native Hawaiian or Other Pacific Islander patients (25%) had the highest ED involvement (vs 23% of non-Hispanic White patients). ED involvement varied by cancer stage at diagnosis (48% distant metastasis, 25% regional metastasis, 10% localized). Patients with a Charlson Comorbidity Index score of 3 or higher had higher levels of ED involvement (36% vs 19% among patients with Charlson Comorbidity Index score of 0). Patients residing in the highest-poverty regions were also more affected (28% vs 20% in the lowest-poverty regions) (Table 2). Among individuals with ED involvement, 77% visited the ED within 7 days of their index date. This proportion varied by tumor site (84% CRC, 78% lung, 60% breast, 54% prostate) and stage at diagnosis (83% distant metastasis, 78% regional metastasis, 64% local) (Figure 1).

**Table 2.** Proportion and characteristics of patients with ED involvement in their diagnosis among SEER-Medicare patients diagnosed with breast, colorectal, lung, or prostate cancer (2008-2017)^a^

	All	ED involvement in diagnosis				ED involvement, by cancer site							
		None		ED involvement		Breast		Colorectal		Lung		Prostate	
						**(n = 139** **068)**		**(n = 111** **955)**		**(n = 186** **348)**		**(n = 177** **377)**	
Characteristic	No.	No.	%	No.	%	No.	%	No.	%	No.	%	No.	%
	614 748	474 060	77.1	140 688	22.9	10 635	7.6	43 474	38.8	74 504	40.0	12 075	6.8
Sex													
Male	320 512	253 930	79.2	66 582	20.8	—	—	18 563	36.1	35 944	39.2	12 075	6.8
Female	294 236	220 130	74.8	74 106	25.2	10 635	7.6	24 911	41.2	38 560	40.7	—	—
Age, y													
66-69	138 295	116 158	84.0	22 137	16.0	1 628	5.1	5170	28.9	13 171	36.6	2168	4.2
70-74	163 396	133 642	81.8	29 754	18.2	2 123	5.9	7 32	31.2	17 618	37.2	2781	4.9
75-79	130 936	101 966	77.9	28 970	22.1	2080	7.3	8185	35.3	16 297	38.2	2408	6.6
80-84	95 362	68 415	71.7	26 947	28.3	1996	9.3	8 32	41.4	13 970	41.9	2049	10.8
≥85	86 759	53 879	62.1	32 880	37.9	2808	13.7	13 955	53.5	13 448	49.7	2669	20.3
Race and ethnicity													
American Indian or Alaska Native	2111	1570	74.4	541	25.6	37	8.4	181	39.3	267	42.7	56	9.6
Asian American	23 138	17 786	76.9	5352	23.1	301	6.2	1918	35.8	2762	38.3	371	6.5
Hispanic	32 080	24 469	76.3	7611	23.7	595	8.5	2661	40.6	3425	46.0	930	8.4
Native Hawaiian or Other Pacific Islander	1533	1148	74.9	385	25.1	39	10.4	97	42.2	207	43.6	42	9.3
Non-Hispanic Black	51 251	37 040	72.3	14 211	27.7	1375	13.4	4276	46.9	6861	51.0	1699	9.2
Non-Hispanic White	498 859	386 657	77.5	112 202	22.5	>8251	>7.2	34 240	38.1	60 873	38.8	>8828	>6.5
Mixed race	298	231	77.5	67	22.5	<11	0.0	20	37.7	35	41.2	<11	0.0
Other/unknown race	5478	5159	94.2	319	5.8	26	4.3	81	20.0	74	32.6	138	3.2
Married or domestic partner													
No	184 182	128 516	69.8	55 666	30.2	5000	9.8	17 708	45.1	29 783	45.2	3175	11.5
Yes	230 216	188 877	82.0	41 339	18.0	2180	4.9	11 791	31.2	22 659	35.1	4709	5.6
Unknown	200 350	156 667	78.2	43 683	21.8	3455	8.0	13 975	40.0	22 062	39.5	4191	6.3
Year of diagnosis													
2008	71 177	55 725	78.3	15 452	21.7	1112	7.8	4964	35.8	8079	38.7	1297	5.9
2009	68 058	53 191	78.2	14 867	21.8	1079	7.6	4657	35.9	7849	38.4	1282	6.2
2010	64 742	50 097	77.4	14 645	22.6	1087	8.0	4610	37.8	7722	39.7	1226	6.3
2011	64 055	49 547	77.4	14 508	22.6	1104	8.0	4501	38.2	7583	40.3	1320	6.7
2012	59 817	45 715	76.4	14 102	23.6	1108	8.0	4323	38.6	7564	40.2	1107	6.9
2013	57 970	44 227	76.3	13 743	23.7	1064	7.7	4164	39.5	7415	40.8	1100	7.1
2014	56 757	43 264	76.2	13 493	23.8	991	7.2	4138	40.2	7262	40.6	1102	7.5
2015	57 449	43 821	76.3	13 628	23.7	1021	7.3	4190	41.3	7234	40.8	1183	7.6
2016	58 056	44 692	77.0	13 364	23.0	1063	7.5	4016	41.6	7057	40.5	1228	7.3
2017	56 667	43 781	77.3	12 886	22.7	1006	7.4	3911	42.2	6739	40.2	1230	7.2
SEER tumor stage													
Localized	307 060	275 697	89.8	31 363	10.2	3761	4.0	12 630	29.8	8625	22.9	6347	4.8
Regional metastasis	130 420	97 708	74.9	32 712	25.1	2642	8.2	16 121	40.3	13 096	31.8	853	5.0
Distant metastasis	142 167	74 512	52.4	67 655	47.6	3676	41.5	11 307	50.5	49 043	50.4	3629	26.7
Unknown	35 101	26 143	74.5	8958	25.5	556	15.4	3416	47.7	3740	36.5	1246	8.9
Charlson Comorbidity Index score													
0	325 185	264 035	81.2	61 150	18.8	5476	6.7	19 996	35.0	29 203	37.8	6475	5.9
1	141 682	109 144	77.0	32 538	23.0	2180	7.0	9479	37.4	18 487	38.1	2392	6.5
2	70 659	51 243	72.5	19 416	27.5	1213	8.8	5661	42.5	11 173	40.8	1369	8.5
≥3	77 222	49 638	64.3	27 584	35.7	1766	13.7	8338	51.6	15 641	47.2	1839	12.2
County geography													
Metropolitan	520 920	402 516	77.3	118 404	22.7	>9111	>7.6	>37 206	>39.4	>62 063	>40.0	>9991	>6.6
Urban, nonmetropolitan	83 507	63 604	76.2	19 903	23.8	1368	8.0	5601	36.0	11 053	40.3	1881	8.1
Rural	10 245	7875	76.9	2370	23.1	145	7.5	656	33.3	1377	38.5	192	6.9
Unknown	76	65	85.5	11	14.5	<11	0.0	<11	0.0	<11	0.0	<11	0.0
Census-tract poverty indicator													
<5% poverty	138 041	111 016	80.4	27 025	19.6	2168	6.4	8938	37.7	13 604	36.6	2315	5.3
5%-10% poverty	156 277	123 139	78.8	33 138	21.2	2491	6.8	10 571	38.0	17 288	38.1	2788	6.0
10%-20% poverty	165 348	126 402	76.4	38 946	23.6	2860	7.8	11 966	38.9	20 779	40.6	3341	7.1
>20% poverty	106 775	77 399	72.5	29 376	27.5	2269	10.3	8795	41.8	15 623	45.4	2689	9.2
Unknown	48 307	36 104	74.7	12 203	25.3	847	8.5	3204	37.3	7210	39.5	942	8.2
SEER registry region													
East	243 682	185 286	76.0	58 396	24.0	4596	8.2	19 319	41.8	29 913	40.3	4568	6.8
South	121 633	93 308	76.7	28 325	23.3	2128	8.4	7591	35.7	16 239	39.5	2367	7.0
Midwest	53 913	40 624	75.4	13 289	24.6	997	8.6	4042	38.8	7198	42.7	1052	7.0
West	195 520	154 842	79.2	40 678	20.8	2914	6.3	12 522	36.7	21 154	39.1	4088	6.7

a ED = emergency department; SEER = Surveillance, Epidemiology, and End Results.

In adjusted models, ED involvement was 3% more prevalent for women overall and for all tumor sites that affect both sexes. Increasing age was associated with ED involvement overall (prevalence ratio = 1.68, 95% CI = 1.66 to 1.71 for patients ≥85 years of age vs patients <70 years of age), with especially notable associations for breast cancer (prevalence ratio = 2.12, 95% CI = 1.99 to 2.25) and prostate cancer (prevalence ratio = 2.66, 95% CI = 2.48 to 2.85). Relative to non-Hispanic White patients, the adjusted prevalence of ED involvement was 30% higher for non-Hispanic Black patients, 22% higher for Native Hawaiian or Other Pacific Islander patients, 17% higher for Hispanic patients, and 16% higher for American Indian or Alaska Native patients. Relative to married or partnered patients, those without a spouse or partner were 23% more likely to have ED involvement, with stronger adjusted associations for patients with breast cancer or prostate cancer. ED involvement increased with recency of year of diagnosis overall (prevalence ratio = 1.16, 95% CI = 1.14 to 1.17 comparing 2016-2017 with 2008-2009) and for patients with CRC, lung cancer, and prostate cancer. Distant-stage cancer was associated with ED involvement overall (prevalence ratio = 2.38, 95% CI = 2.34 to 2.41), with substantial variation by site (breast cancer prevalence ratio = 8.95, 95% CI = 8.57 to 9.35; CRC prevalence ratio = 1.67, 95% CI = 1.65 to 1.71; lung cancer prevalence ratio = 2.04, 95% CI = 2.00 to 2.08; prostate cancer prevalence ratio = 3.83, 95% CI = 3.65 to 4.01). Relative to the lowest comorbidity scores, patients with the highest comorbidity scores had 21% higher adjusted prevalence of ED involvement overall and for CRC, lung cancer, and breast cancer, but comorbidity status was not associated with ED involvement for patients with prostate cancer. Compared with the largest metropolitan areas, residing in smaller metropolitan and nonmetropolitan counties was associated with ED involvement for patients with prostate cancer and breast cancer. Patients residing in higher census-tract–level poverty areas were 18% more likely to have ED involvement (vs patients in the lowest poverty group), with stronger associations for patients with prostate cancer and breast cancer (Table 3).

**Table 3.** Associations between patient sociodemographic, clinical, and tumor characteristics and ED involvement among SEER-Medicare patients diagnosed with breast, colorectal, lung, or prostate cancer (2008-2017)

Characteristic	All cancer sites, prevalence ratio (95% CI)	Breast, prevalence ratio (95% CI)	Colorectal, prevalence ratio (95% CI)	Lung, prevalence ratio (95% CI)	Prostate, prevalence ratio (95% CI)
	**(N = 530** **138)**	**(n = 125** **201)**	**(n = 96** **379)**	**(n = 158** **661)**	**(n = 149** **897)**
Sex^a^					
Male	[Referent]	N/A	[Referent]	[Referent]	—
Female	1.03 (1.02 to 1.03)	—	1.03 (1.02 to 1.05)	1.03 (1.02 to 1.04)	N/A
Age, y^a^					
66-69	[Referent]	[Referent]	[Referent]	[Referent]	[Referent]
70-74	1.07 (1.05 to 1.08)	1.12 (1.05 to 1.19)	1.07 (1.04 to 1.10)	1.02 (1.00 to 1.04)	1.18 (1.11 to 1.25)
75-79	1.18 (1.17 to 1.20)	1.34 (1.26 to 1.43)	1.22 (1.18 to 1.25)	1.07 (1.05 to 1.09)	1.45 (1.36 to 1.54)
80-84	1.36 (1.34 to 1.39)	1.62 (1.52 to 1.73)	1.42 (1.38 to 1.46)	1.17 (1.15 to 1.19)	1.98 (1.86 to 2.12)
≥85	1.68 (1.66 to 1.71)	2.12 (1.99 to 2.25)	1.75 (1.70 to 1.79)	1.37 (1.35 to 1.40)	2.66 (2.48 to 2.85)
Race and ethnicity^a^					
American Indian or Alaska Native	1.16 (1.08 to 1.24)	1.30 (0.96 to 1.76)	1.10 (0.99 to 1.23)	1.12 (1.03 to 1.22)	1.26 (0.97 to 1.64)
Asian American	0.99 (0.97 to 1.01)	1.09 (0.98 to 1.22)	1.01 (0.98 to 1.05)	0.98 (0.95 to 1.00)	1.02 (0.92 to 1.14)
Hispanic	1.17 (1.15 to 1.19)	1.26 (1.17 to 1.37)	1.11 (1.08 to 1.15)	1.15 (1.13 to 1.18)	1.40 (1.30 to 1.50)
Native Hawaiian or Other Pacific Islander	1.23 (1.13 to 1.32)	1.65 (1.25 to 2.18)	1.26 (1.09 to 1.47)	1.11 (1.01 to 1.22)	1.55 (1.15 to 2.09)
Non-Hispanic Black	1.30 (1.28 to 1.32)	1.54 (1.45 to 1.63)	1.25 (1.22 to 1.28)	1.24 (1.22 to 1.26)	1.52 (1.43 to 1.60)
Non-Hispanic White	[Referent]	[Referent]	[Referent]	[Referent]	[Referent]
Mixed race	1.12 (0.93 to 1.34)	1.41 (0.69 to 2.87)	1.08 (0.75 to 1.55)	1.11 (0.89 to 1.37)	1.06 (0.49 to 2.27)
Married or domestic partner^b^					
Yes	[Referent]	[Referent]	[Referent]	[Referent]	[Referent]
No	1.23 (1.22 to 1.25)	1.35 (1.28 to 1.42)	1.22 (1.20 to 1.25)	1.17 (1.16 to 1.19)	1.45 (1.38 to 1.52)
Unknown	1.09 (1.07 to 1.10)	1.25 (1.17 to 1.33)	1.08 (1.05 to 1.10)	1.07 (1.05 to 1.08)	1.09 (1.03 to 1.14)
Year of diagnosis^a^					
2008-2009	[Referent]	[Referent]	[Referent]	[Referent]	[Referent]
2010-2011	1.05 (1.04 to 1.07)	1.07 (1.01 to 1.13)	1.07 (1.04 to 1.09)	1.05 (1.03 to 1.07)	1.07 (1.01 to 1.13)
2012-2013	1.09 (1.08 to 1.11)	1.04 (0.98 to 1.11)	1.12 (1.09 to 1.14)	1.08 (1.06 to 1.10)	1.12 (1.05 to 1.19)
2014-2015	1.11 (1.10 to 1.13)	0.99 (0.94 to 1.05)	1.17 (1.15 to 1.20)	1.09 (1.07 to 1.11)	1.13 (1.07 to 1.20)
2016-2017	1.16 (1.14 to 1.17)	1.04 (0.98 to 1.11)	1.20 (1.18 to 1.24)	1.14 (1.12 to 1.16)	1.13 (1.06 to 1.20)
SEER tumor stage^a^					
Localized	[Referent]	[Referent]	[Referent]	[Referent]	[Referent]
Regional metastasis	1.48 (1.46 to 1.51)	1.93 (1.84 to 2.03)	1.29 (1.27 to 1.31)	1.35 (1.31 to 1.38)	1.13 (1.05 to 1.21)
Distant metastasis	2.38 (2.35 to 2.41)	8.97 (8.59 to 9.37)	1.68 (1.65 to 1.71)	2.03 (1.99 to 2.07)	3.85 (3.67 to 4.04)
Charlson Comorbidity Index score^c^					
0	[Referent]	[Referent]	[Referent]	[Referent]	[Referent]
1	1.07 (1.05 to 1.08)	1.01 (0.97 to 1.07)	1.07 (1.05 to 1.09)	1.07 (1.05 to 1.08)	0.97 (0.92 to 1.02)
2	1.15 (1.13 to 1.16)	1.12 (1.05 to 1.20)	1.17 (1.14 to 1.20)	1.13 (1.11 to 1.15)	1.02 (0.95 to 1.09)
≥3	1.21 (1.19 to 1.23)	1.19 (1.11 to 1.28)	1.26 (1.22 to 1.29)	1.19 (1.17 to 1.21)	0.97 (0.91 to 1.05)
County geography^d^					
Metropolitan	[Referent]	[Referent]	[Referent]	[Referent]	[Referent]
Urban, nonmetropolitan	1.03 (1.01 to 1.04)	1.09 (1.02 to 1.15)	0.97 (0.95 to 0.99)	1.02 (1.00 to 1.04)	1.23 (1.16 to 1.30)
Rural	0.91 (0.87 to 0.96)	0.93 (0.76 to 1.14)	0.82 (0.75 to 0.90)	0.96 (0.91 to 1.02)	0.98 (0.81 to 1.18)
Census-tract poverty indicator^e^					
<5% poverty	[Referent]	[Referent]	[Referent]	[Referent]	[Referent]
5%-10% poverty	1.04 (1.02 to 1.05)	1.01 (0.96 to 1.07)	1.02 (1.00 to 1.04)	1.03 (1.02 to 1.05)	1.10 (1.04 to 1.16)
10%-20% poverty	1.11 (1.09 to 1.12)	1.12 (1.06 to 1.18)	1.06 (1.04 to 1.09)	1.10 (1.08 to 1.12)	1.19 (1.12 to 1.26)
>20% poverty	1.18 (1.16 to 1.20)	1.23 (1.15 to 1.30)	1.10 (1.07 to 1.13)	1.16 (1.14 to 1.18)	1.39 (1.30 to 1.48)
SEER registry region^a^					
East	1.05 (1.04 to 1.07)	1.15 (1.10 to 1.20)	1.08 (1.06 to 1.10)	1.02 (1.01 to 1.03)	0.97 (0.93 to 1.02)
Midwest	1.02 (1.01 to 1.04)	1.15 (1.07 to 1.23)	0.98 (0.95 to 1.01)	1.02 (1.00 to 1.04)	1.03 (0.96 to 1.10)
South	0.99 (0.97 to 1.00)	1.14 (1.08 to 1.22)	0.94 (0.92 to 0.97)	0.98 (0.96 to 0.99)	1.01 (0.95 to 1.07)
West	[Referent]	[Referent]	[Referent]	[Referent]	[Referent]

a Prevalence ratios estimated using generalized linear models with log link and Poisson distribution. Model 1 independent variables included age group; sex; race category; sex; SEER region; disease stage at diagnosis; year of diagnosis; source of diagnosis date (registry vs claims); count of outpatient, inpatient, and emergency department visit days in the year before diagnosis (as 3 separate variables); and tumor site (for all cancers model only). ED = emergency department; N/A = not applicable; SEER = Surveillance, Epidemiology, and End Results.

b Model 3 include model 2 variables and marital status.

c Model 5 includes model 4 variables and Charlson Comorbidity Index.

d Model 2 includes model 1 variables and county geography.

e Model 4 includes model 3 variables and poverty category.

When we restricted the analysis to patients with an exact index date, our results were materially identical to the size, direction, and significance of main analysis estimates. (Supplementary Tables 1 and 2, available online).

## Discussion

Our results indicate that about 4 in 10 patients with CRC or lung cancer and 1 in 15 patients with breast cancer or prostate cancer may have visited an ED at the beginning of their diagnostic episode, with the majority visiting the ED in the 7 days before the first indication of cancer appeared in their medical records. We also found that non-Hispanic Black patients, Hispanic patients, and Native Hawaiian or Other Pacific Islander patients; individuals who are older, unpartnered, or residing in lower-income neighborhoods; and those patients with more comorbidities were more likely to have ED involvement in their cancer diagnosis.

Our estimates are consistent with recent international studies of emergency presentation for cancer. In an analysis of data from 14 jurisdictions in 6 countries, the prevalence of emergency presentation ranged from 26% to 51% for lung cancer, 23% to 37% for colon cancer, and 9% to 20% for rectal cancer (3). Furthermore, a 2019 UK study found that between 2006 and 2013, 5% of patients with breast cancer, 11% of patients with prostate cancer, 16% of patients with rectal cancer, 34% of patients with colon cancer, and 39% of patients with lung cancer were emergency presentations (5). In the United States, Pettit et al. recently reported that 19% of patients with lung cancer, 9% of patients with breast cancer, 9% of patients with CRC, and 6% of patients with prostate cancer diagnosed statewide in Indiana (2013-2017) had evidence of ED utilization in the 6 months before diagnosis (10). Our results may differ from theirs because of differences in Indiana (which is not a SEER registry), the older age distribution of our sample, or other methodological differences. Other recent US studies were from single safety-net hospitals in New York City (2012-2014)—where 42% of 638 patients had CRC diagnosed through the ED (11)—and in Jacksonville, Florida (2009-2011)—where 32% of all patients with cancer (N = 989) were admitted through the ED (12). Our results also update population-based estimates by Pruitt et al. (7), who observed that 29% of patients with CRC in SEER-Medicare (1992-2005) had an ED claim related to obstruction, perforation, or emergency inpatient admission in the same month as their registry date of diagnosis.

The prevalence of ED involvement varied substantially by tumor site and was more common in later-stage tumors. This finding aligns with previous literature (24). Although all 4 cancer types in this study were detectable through guideline-recommended screening tests during the study period, screening adherence was higher for breast and prostate cancers, which had the lowest levels of ED involvement, and are also more often detected at earlier stages. Even in the absence of screening, tumors of the breast and prostate have specific (and often less medically acute) symptomology that can facilitate earlier, nonemergent diagnosis, such as breast lump or hematuria, respectively. Patients with lung cancer and CRC had the highest levels of ED involvement. In the absence of screening, lung cancer and CRC often progress unnoticed until they produce a dramatic and medically serious event, such as intestinal perforation, or “red flag” symptoms, such as hemoptysis or hematochezia, the experience of which may precipitate a patient visit to the ED.

Older age and the presence of multiple comorbidities were associated with ED involvement, a finding consistent with prior literature (24). Older age is associated with functional decline and cognitive impairment, which may affect a patient’s ability to recognize and report cancer symptoms, or with social isolation, limiting the social and economic support needed for attending ambulatory preventive care services (25). Morbidities may make attribution of symptoms of underlying cancer harder and may also distract attention from investigation of new symptoms (26). In addition, older age increases the risk of frailty, which can increase the risk of falls and accidents that may lead to incidental diagnosis of cancer, as well as comorbidities, such that age and comorbidity may represent compounding influences that can complicate or delay cancer diagnosis (27,28).

Unmarried or unpartnered marital status was associated with ED involvement, as well. This finding has not been widely reported beyond 2 prior Norwegian studies (29,30). Although some emergency diagnoses may be considered unavoidable because of tumor biology (24) or driven primarily by symptomology (31), the increased risk of ED involvement for unpartnered patients observed across all tumor sites studied suggest the importance of nonbiological drivers of emergency diagnosis. Being married or partnered is associated with earlier stage at diagnosis (32) and improved patient survival (33), and this relationship is largely attributed to social support. Potential explanations for the relationship with emergency presentation include previous observations that unmarried patients are less likely to have a usual source of care (34) or to seek medical care for nonspecific symptoms (35).

We observed statistically significant adjusted associations between patient race, socioeconomic conditions and ED involvement, with a higher prevalence of ED involvement among patients who were non-Hispanic Black, Native Hawaiian or Other Pacific Islander, and living in regions with more than 20% poverty. These relationships were observed in previous US studies (6,7,10) and contribute to a wide body of literature describing disparities in burden and outcomes across the care continuum for patients with cancer in the United States. This relationship, which persisted after adjusting for tumor stage and other covariates, also points to nonbiological drivers of ED involvement and may be explained by higher ED utilization for routine care (36) or poor-quality primary care (37). These populations are also known to have lower-quality cancer care and worse outcomes independent of treatment. With regard to these outcome disparities, there may be a compounding effect of ED involvement, as it has also been found to be associated with reduced receipt of curative treatment (24). The finding of higher ED involvement among Native Hawaiian or Other Pacific Islander populations is novel and underlines the importance of separating these patients from the larger population of Asian Americans, whose levels of ED involvement were similar to non-Hispanic White patients (38).

This large, nationally representative study used high-quality data. Medicare patients are insured and should have access to primary and specialty care. Our findings, however, may not be generalizable beyond the US Medicare fee-for-service population. We were not able to include a growing portion of beneficiaries who participate in Medicare Advantage plans or patients insured only under Medicaid or other state or federal insurance systems (eg, US Department of Veterans Affairs) or the uninsured. These excluded populations may differ from our study population in terms of demographics, health-care access, cancer burden (39-42), and (consequently) ED involvement. Barriers to care are often higher among uninsured and younger individuals, so the estimates presented here may be attenuated compared with the general population; this hypothesis is corroborated by reports of higher levels of emergency presentation than we observed in safety-net populations (11). Our use of disease stage at diagnosis as a proxy for extent of disease at presentation is imperfect and may be confounded by an unobserved third variable (ie, health-care seeking preferences, quality of preventive care) that may be correlated with both delay in cancer detection and the propensity to visit the ED. Our use of a claims-based index date improves upon prior research anchoring on the cancer registration date of diagnosis, which is inconsistently defined, and may not reflect the initiation of cancer-relevant diagnostic health care (43) when ED utilization would be indicative of its involvement in identifying the cancer. In the absence of chart review, however, which would have been infeasible given the population-based nature of the study and sample size, it is impossible to know with certainty that ED clinicians played a role in the identification of a patient’s cancer. It is possible that patients coincidentally visited the ED within 30 days of cancer identification, which would result in overestimation of ED involvement, or underestimation occurred by classifying as non-ED involved those patients who visited the ED in the days immediately following their index date. Nonetheless, the degree of misclassification that would have been required to explain the observed patterns of large variation in prevalence of ED involvement (eg, by disease stage and by cancer site) would have had to be implausibly large. Furthermore, our findings regarding tumor and patient factors associated with ED involvement concord fully with recent international and prior US studies, and they provide a timely update to an understudied topic in US cancer population research.

Even for cancers with available screening modalities and in a population with health-care coverage, a substantial proportion of patients visit the ED in the days and weeks before they are initially diagnosed with cancer, and ED clinicians may be the first to identify or suspect and coordinate workup of up to 40% of colorectal and lung cancers. Considering the burden of CRC and lung cancer, these results suggest that the ED plays an important role in cancer diagnosis in the United States. Patients whose cancer diagnosis starts or originates in the ED may reflect a constellation of factors, many of which are potentially modifiable at the patient and system levels, including insufficient awareness of prodromal symptoms of cancer or delayed help seeking, a failure of access to screening, suboptimal access and quality of the primary health-care system, and barriers to ambulatory investigations (8). The barriers to nonemergency diagnosis of cancer may be particularly applicable to populations historically targeted for marginalization, such as racial and ethnic minority groups, or those individuals living in lower socioeconomic neighborhoods and rural areas. Emergency diagnosis may also reflect or exaggerate known racial, ethnic, and socioeconomic disparities in cancer outcomes due to delayed treatment, undertreatment, and suboptimal access to preventive care and screening. System-level efforts to improve the identification of cancer in non-ED settings, before the patient reaches the ED, may improve patient outcomes and health-care system efficiency. Further research is needed to better understand how emergency diagnosis is related to survival, quality of care, and patient experience in US populations.

## Supplementary Material

pkae039_Supplementary_Data

## Data Availability

Although the SEER-Medicare data are not public use data files, investigators can obtain all data necessary to reproduce the current analysis by request to the National Cancer Institute Division of Cancer Control and Population Sciences. Procedures to obtain approval and acquire the data are provided on its website (https://healthcaredelivery.cancer.gov/seermedicare/obtain/).
