# Supplementary material for: Emergency department involvement in the diagnosis of cancer among older adults: a SEER-Medicare study
Source: JNCI Cancer Spectr. 2024 May 25;8(3):pkae039. doi: 10.1093/jncics/pkae039 (PMC11193434; doi:10.1093/jncics/pkae039)
Supplement: pkae039_Supplementary_Data [file pkae039_supplementary_data.docx]

**Supplementary Figure 1: Study sample flow diagram**


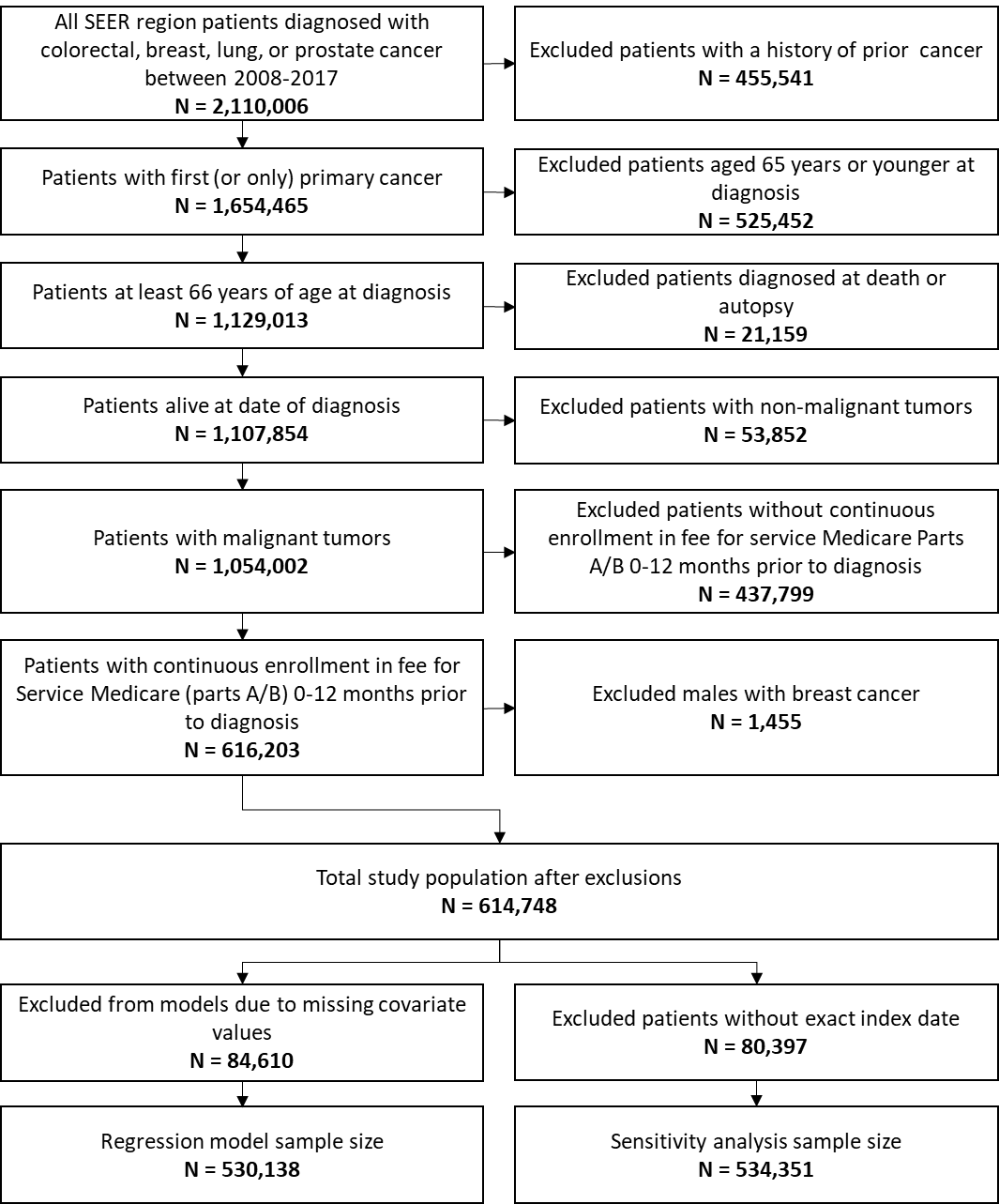


**Supplementary Methods**

Model building strategy:

1. We included the following adjustment variables in all models listed below. Our decision to force these into the model was based on background knowledge and approaches in existing literature; we have provided a brief rationale for each.
   1. *Age at diagnosis*: age is strongly associated with cancer incidence and myriad other social and health-related factors, it is universally adjusted in studies of cancer.
   2. *Year of diagnosis*: cancer incidence decreased over the study period and emergency room visits increased, also some SEER registries did not contribute case counts for all years of data.
   3. *Stage at diagnosis*: while stage is not recorded until after the diagnosis, it exists prior to the diagnosis and is measured around the time of diagnosis; we include it as a proxy of the disease spread just prior to diagnosis, which we anticipate will be highly correlated with emergencies.
   4. *Counts of inpatient-, outpatient- and emergency department- visit-days in the year prior to diagnosis*: to adjust for underlying rates of healthcare utilization (HCU), which will explain variation in cancer occurrence, stage at diagnosis, and ED utilization.
   5. *SEER registry and region*: fixed effect for clustered data.
   6. *Imputed vs. exact index date*: to account for outcome classification error, which might be more common for patients with imputed index dates.
   7. *Tumor site*: only for models that include all cancer sites.
2. For all additional exposures (race and ethnicity, marital status, county rural/urban status, and census tract poverty) and comorbidity score, we used a DAG (and dagitty.net) to determine our sufficient adjustment set.
3. This DAG, without a defined exposure and no adjustments is pictured below.
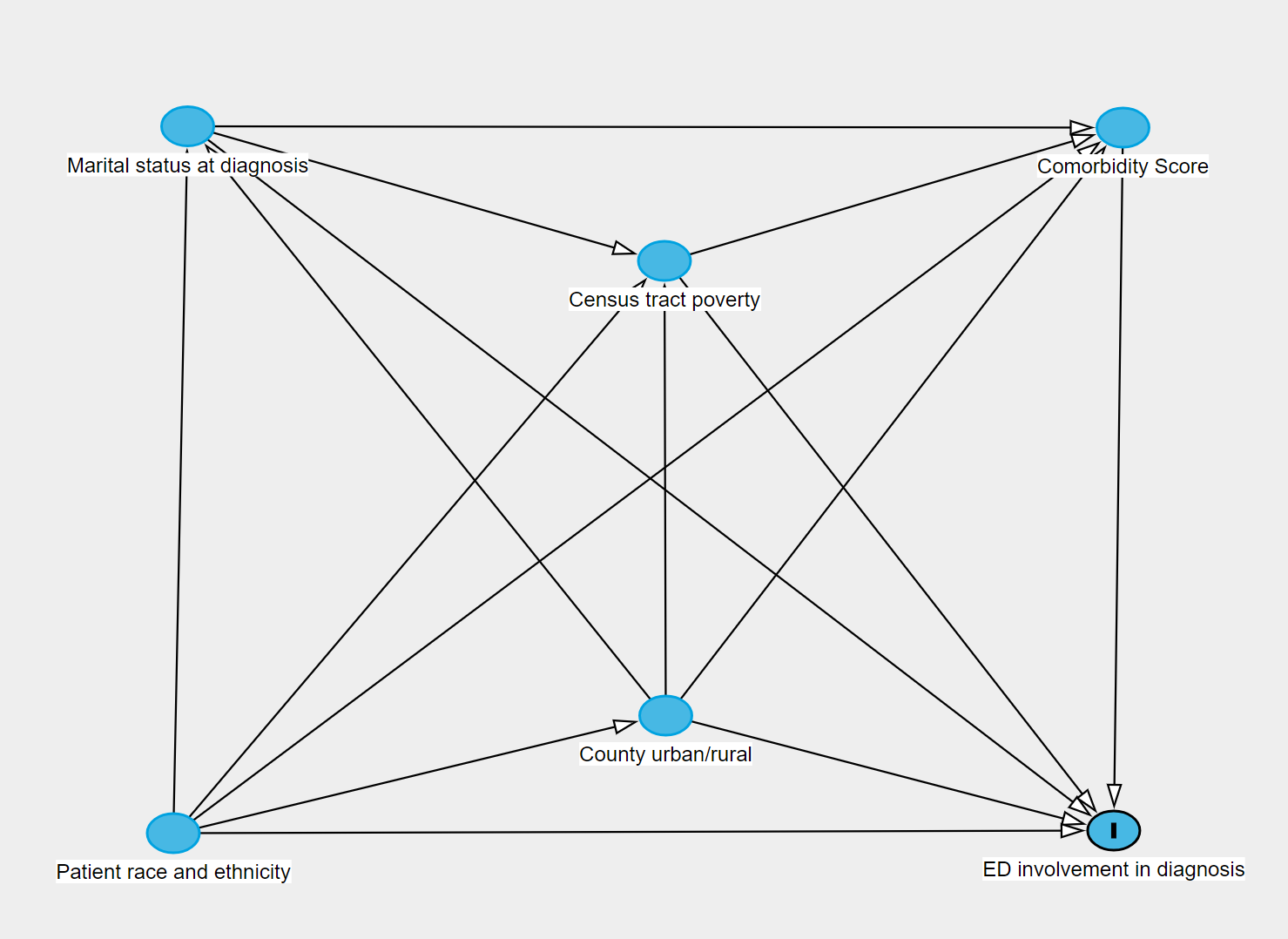

4. Adjustment sets considering each additional variable as the exposure (one exposure at a time), are explained in the table.

| With patient race/ethnicity as the exposure, no additional adjustments are necessary.  This is model 1. | 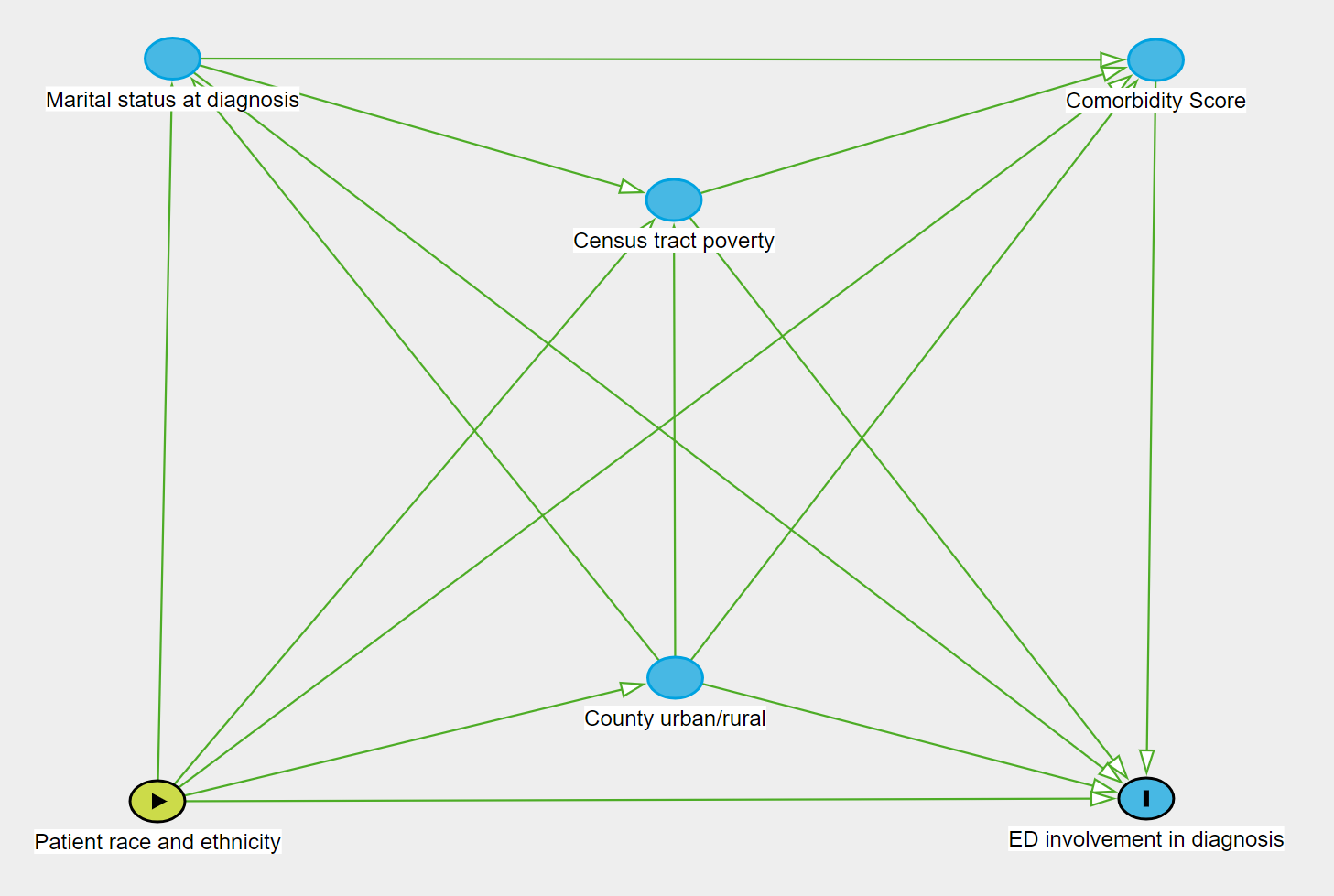 |
| --- | --- |
| With county urban/rural status as the exposure, race/ethnicity must be adjusted.  This is model 2. | 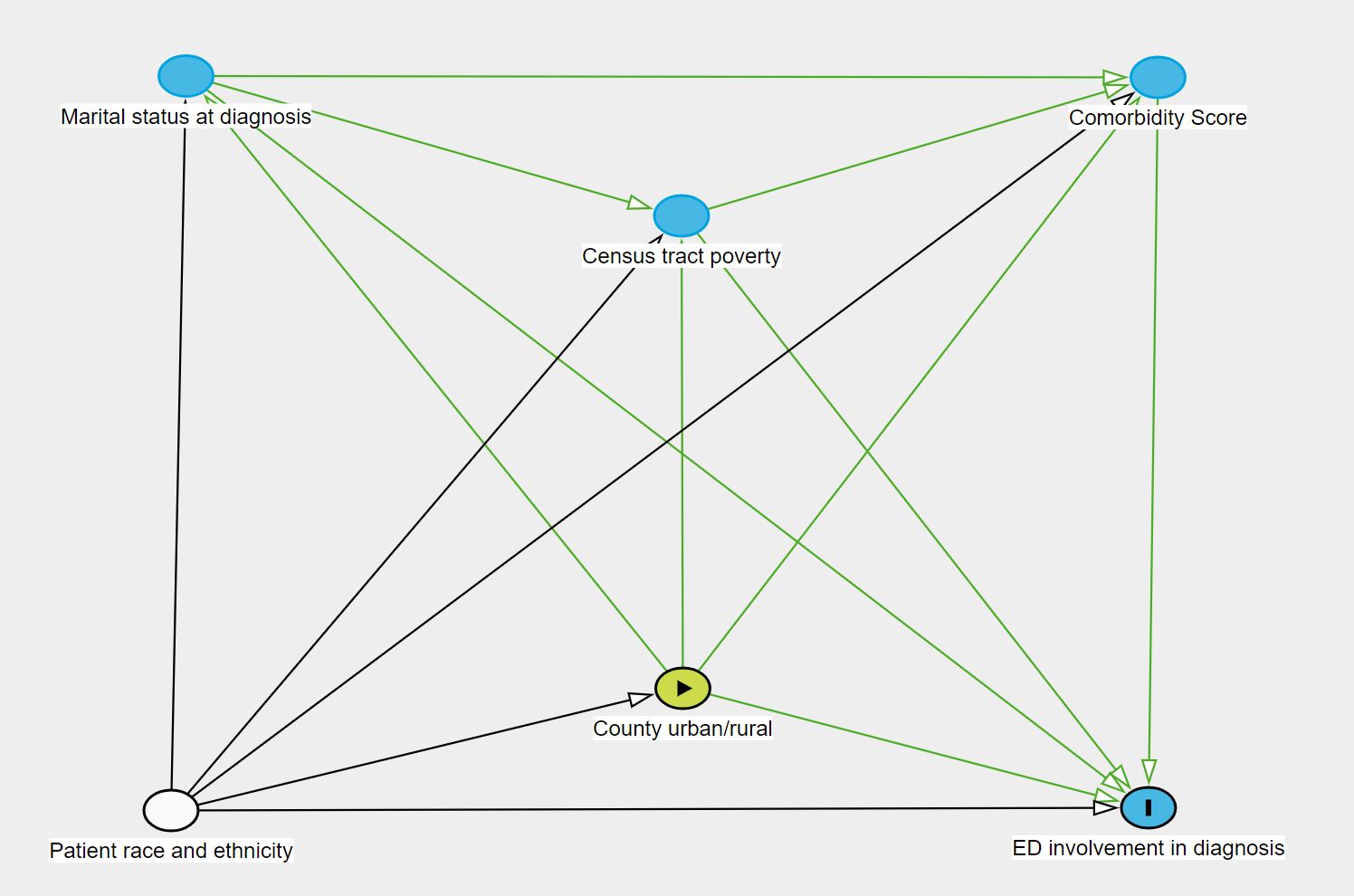 |
| With marital status as the exposure, race/ethnicity and county urban/rural status must be adjusted.  This is model 3. | 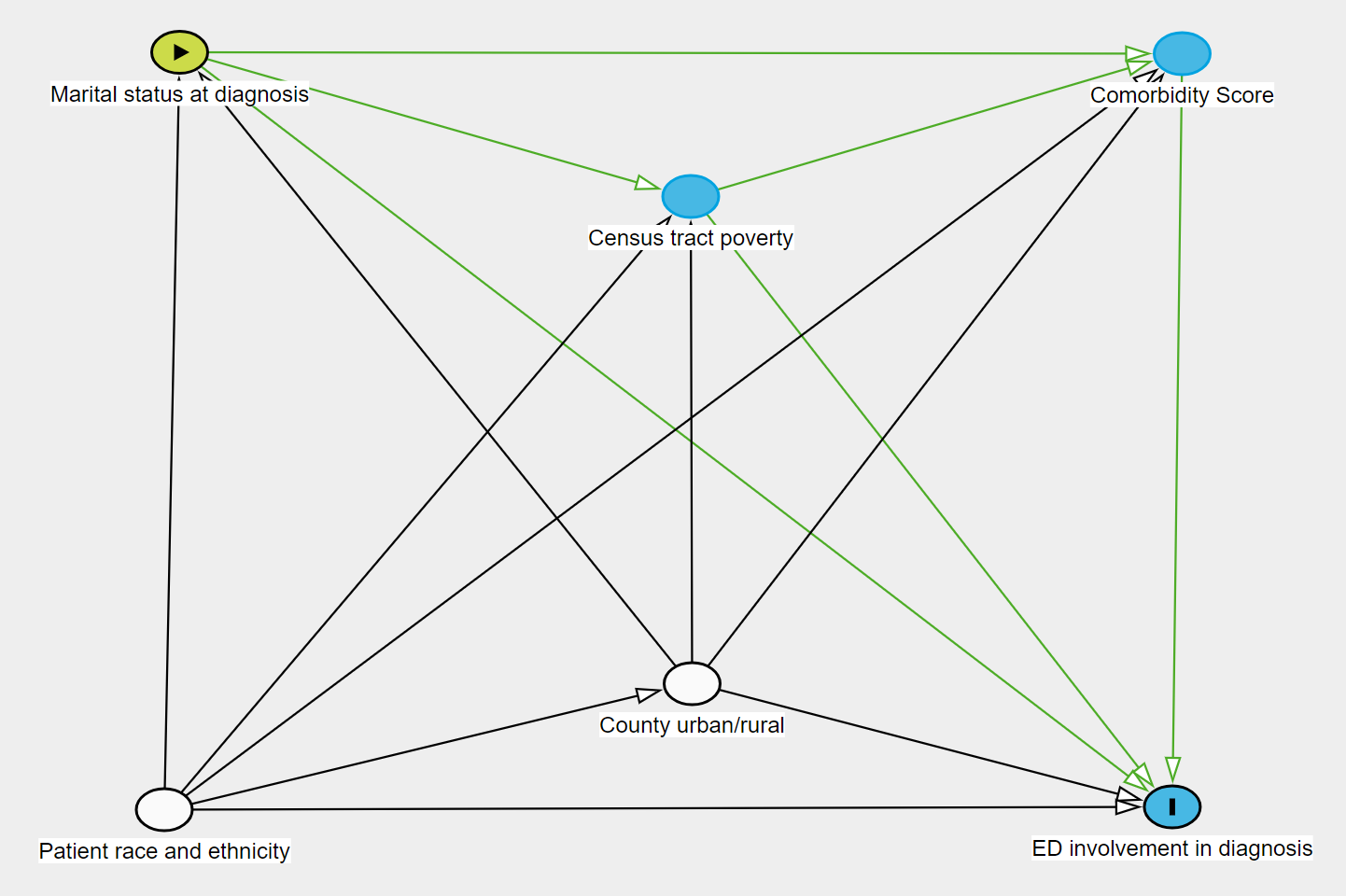 |
| With census tract level poverty as the exposure, race/ethnicity, county urban/rural status, and marital status must be adjusted.  This is model 4. | 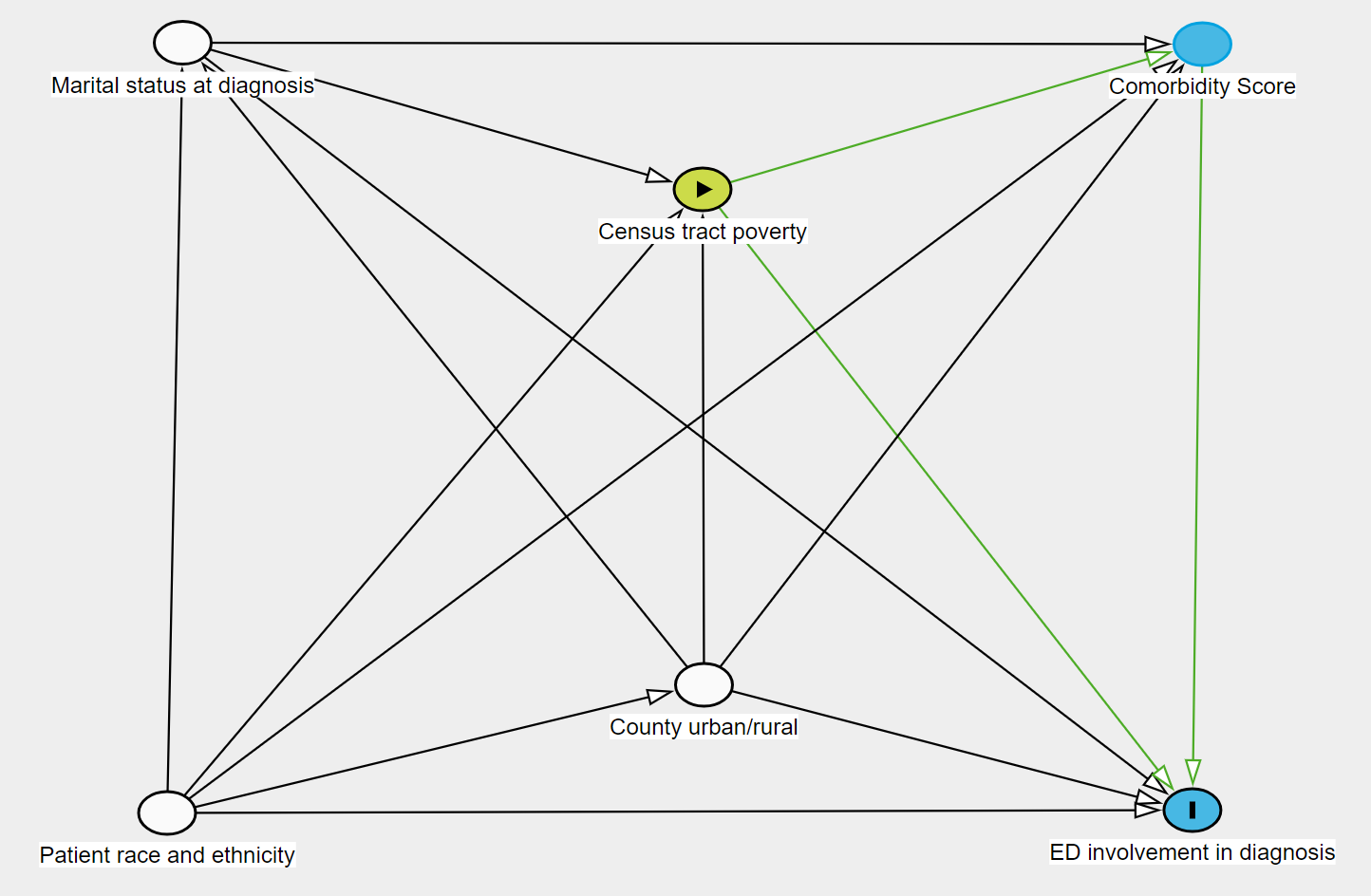 |
| With comorbidity as the exposure, race category, county urban/rural, marital status, and poverty must be adjusted.  This is model 5. | 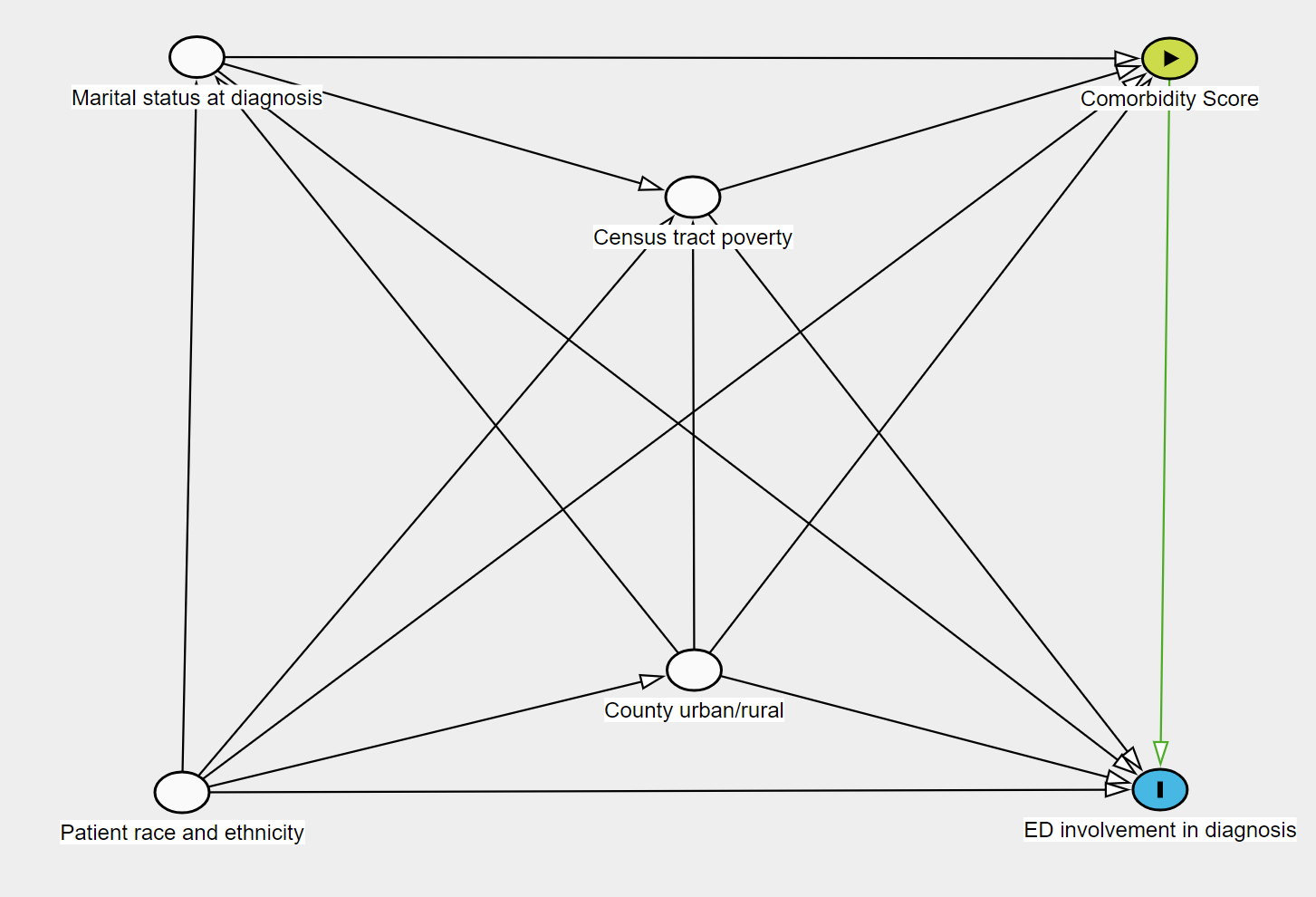 |

**Supplementary Table 1.** Proportion and characteristics of patients with emergency department (ED) involvement in their diagnosis among SEER-Medicare patients diagnosed with breast, colorectal, lung, and prostate cancers (2008-2017), excluding patients with imputed index dates

|  | **All** | **ED Involvement in Diagnosis, row %** | | | | **% with ED Involvement by Cancer Site** | | | | | | | |
| --- | --- | --- | --- | --- | --- | --- | --- | --- | --- | --- | --- | --- | --- |
|  |  | **None** | | **ED Involvement** | | **Breast** | | **Colorectal** | | **Lung** | | **Prostate** | |
|  | **N** | **N** | **%** | **N** | **%** | **N** | **%** | **N** | **%** | **N** | **%** | **N** | **%** |
| **All** | 534,351 | 403,674 | 75.5 | 130,677 | 24.5 | 9,580 | 7.9 | 41,813 | 41.6 | 67,290 | 46.7 | 11,994 | 7.1 |
| **Sex** |  |  |  |  |  |  |  |  |  |  |  |  |  |
| Male | 283,663 | 221,270 | 78.0 | 62,393 | 22.0 | - | - | 17,838 | 39.2 | 32,561 | 46.8 | 11,994 | 7.1 |
| Female | 250,688 | 182,404 | 72.8 | 68,284 | 27.2 | 9,580 | 7.9 | 23,975 | 43.5 | 34,729 | 46.6 | - | - |
| **Age in Years** |  |  |  |  |  |  |  |  |  |  |  |  |  |
| 66-69 years | 120,334 | 99,738 | 82.9 | 20,596 | 17.1 | 1,488 | 5.3 | 4,926 | 31.6 | 12,040 | 43.0 | 2,142 | 4.4 |
| 70-75 years | 169,005 | 116,063 | 80.7 | 27,756 | 19.3 | 1,938 | 6.1 | 6,966 | 33.5 | 16,088 | 43.3 | 2,764 | 5.1 |
| 76-80 years | 107,697 | 87,571 | 76.5 | 26,950 | 23.5 | 1,866 | 7.4 | 7,898 | 37.6 | 14,793 | 44.4 | 2,393 | 6.8 |
| 81-85 years | 76,704 | 57,313 | 69.5 | 25,100 | 30.5 | 1,796 | 9.6 | 8,616 | 43.9 | 12,643 | 49.0 | 2,045 | 11.2 |
| >85 years | 60,611 | 42,989 | 58.7 | 30,275 | 41.3 | 2,492 | 14.3 | 13,407 | 56.9 | 11,726 | 58.9 | 2,650 | 21.3 |
| **Race and Ethnicity** |  |  |  |  |  |  |  |  |  |  |  |  |  |
| Non-Hispanic White (NHW) | 434,538 | 330,490 | 76.1 | 104,048 | 23.9 | >7,425 | >7.4 | 32,981 | 40.6 | 54,859 | 45.3 | >8,772 | >6.7 |
| Non-Hispanic Black (NHB) | 43,491 | 30,282 | 69.6 | 13,209 | 30.4 | 1,226 | 14.3 | 4,083 | 51.7 | 6,216 | 59.6 | 1,684 | 10.2 |
| Hispanic | 27,935 | 20,797 | 74.4 | 7,138 | 25.6 | 540 | 9.1 | 2,557 | 44.1 | 3,117 | 53.2 | 924 | 8.9 |
| Asian American | 19,976 | 14,918 | 74.7 | 7,138 | 25.6 | 283 | 6.8 | 1,830 | 38.9 | 2,577 | 45.3 | 368 | 6.8 |
| American Indian or Alaskan Native (AIAN) | 1,750 | 1,253 | 71.6 | 497 | 28.4 | 34 | 9.2 | 175 | 43.6 | 233 | 50.9 | 55 | 10.5 |
| Native Hawaiian or Other Pacific Islander (NHOPI) | 1,279 | 922 | 72.1 | 357 | 27.9 | 36 | 10.7 | 92 | 47.4 | 187 | 53.0 | 42 | 10.6 |
| Mixed Race | 252 | 188 | 74.6 | 64 | 25.4 | <11 | 0.0 | 19 | 42.2 | 34 | 54.0 | <11 | 0.0 |
| Other/Unknown Race | 5,130 | 4,824 | 94.0 | 306 | 6.0 | 25 | 4.8 | 76 | 23.8 | 67 | 41.1 | 138 | 3.3 |
| **Married or Domestic Partner** |  |  |  |  |  |  |  |  |  |  |  |  |  |
| Yes | 203,151 | 164,511 | 81.0 | 38,640 | 19.0 | 2,006 | 5.1 | 11,367 | 33.5 | 20,590 | 41.0 | 4,677 | 5.9 |
| No | 155,489 | 104,191 | 67.0 | 51,298 | 33.0 | 4,486 | 10.1 | 16,967 | 48.1 | 26,692 | 52.7 | 3,153 | 12.5 |
| Unknown | 175,711 | 134,972 | 76.8 | 40,739 | 23.2 | 3,088 | 8.3 | 13,479 | 43.0 | 20,008 | 46.1 | 4,164 | 6.5 |
| **Year of Diagnosis** |  |  |  |  |  |  |  |  |  |  |  |  |  |
| 2008 | 61,946 | 47,495 | 76.7 | 14,451 | 23.3 | 981 | 8.1 | 4,806 | 38.3 | 7,378 | 45.0 | 1,286 | 6.2 |
| 2009 | 59,265 | 45,373 | 76.6 | 13,892 | 23.4 | 965 | 8.0 | 4,489 | 38.5 | 7,166 | 44.5 | 1,272 | 6.6 |
| 2010 | 56,282 | 42,610 | 75.7 | 13,672 | 24.3 | 962 | 8.2 | 4,454 | 40.7 | 7,039 | 46.3 | 1,217 | 6.6 |
| 2011 | 56,111 | 42,528 | 75.8 | 13,583 | 24.2 | 997 | 8.3 | 4,340 | 40.7 | 6,934 | 46.8 | 1,312 | 7.0 |
| 2012 | 51,972 | 38,845 | 74.7 | 13,127 | 25.3 | 1,008 | 8.3 | 4,145 | 41.1 | 6,879 | 46.9 | 1,095 | 7.2 |
| 2013 | 50,291 | 37,544 | 74.7 | 12,747 | 25.3 | 960 | 8.0 | 4,005 | 42.3 | 6,688 | 47.4 | 1,094 | 7.5 |
| 2014 | 49,118 | 36,671 | 74.7 | 12,447 | 25.3 | 897 | 7.4 | 3,967 | 42.9 | 6,484 | 47.4 | 1,099 | 7.8 |
| 2015 | 49,780 | 37,194 | 74.7 | 12,586 | 25.3 | 923 | 7.5 | 4,007 | 44.1 | 6,481 | 47.8 | 1,175 | 7.9 |
| 2016 | 50,354 | 38,045 | 75.6 | 12,309 | 24.4 | 969 | 7.8 | 3,851 | 44.6 | 6,270 | 47.8 | 1,219 | 7.6 |
| 2017 | 49,232 | 37,369 | 75.9 | 11,863 | 24.1 | 918 | 7.6 | 3,749 | 45.0 | 5,971 | 47.6 | 1,225 | 7.5 |
| **SEER Tumor Stage** |  |  |  |  |  |  |  |  |  |  |  |  |  |
| Localized | 275,409 | 246,364 | 89.5 | 29,045 | 10.5 | 3,329 | 4.0 | 12,203 | 31.9 | 7,203 | 25.8 | 6,310 | 5.0 |
| Regional | 114,905 | 83,851 | 73.0 | 31,054 | 27.0 | 2,436 | 8.6 | 15,884 | 42.1 | 11,886 | 36.5 | 848 | 5.2 |
| Distant | 117,915 | 54,835 | 46.5 | 63,080 | 53.5 | 3,406 | 45.4 | 10,668 | 55.4 | 45,393 | 58.0 | 3,613 | 27.9 |
| Unknown | 26,122 | 18,624 | 71.3 | 7,498 | 28.7 | 409 | 17.9 | 3,058 | 57.1 | 2,808 | 51.1 | 1,223 | 9.4 |
| **Charlson Comorbidity Score (0-3+)** |  |  |  |  |  |  |  |  |  |  |  |  |  |
| 0 | 281,846 | 224,338 | 79.6 | 57,508 | 20.4 | 5,047 | 7.1 | 19,283 | 38.1 | 26,761 | 45.7 | 6,417 | 6.3 |
| 1 | 124,365 | 94,225 | 75.8 | 30,140 | 24.2 | 1,937 | 7.2 | 9,113 | 39.4 | 16,710 | 43.8 | 2,380 | 6.6 |
| 2 | 61,563 | 43,589 | 70.8 | 17,974 | 29.2 | 1,075 | 9.0 | 5,471 | 45.1 | 10,062 | 46.7 | 1,366 | 8.6 |
| 3+ | 66,577 | 41,522 | 62.4 | 25,055 | 37.6 | 1,521 | 13.7 | 7,946 | 53.9 | 13,757 | 53.0 | 1,831 | 12.4 |
| **County Geography** |  |  |  |  |  |  |  |  |  |  |  |  |  |
| Metropolitan | 454,068 | 343,645 | 75.7 | 110,423 | 24.3 | >8,248 | >7.9 | >35,845 | >42.3 | >56,394 | >46.6 | >9,923 | >6.9 |
| Urban, non-metropolitan | 71,470 | 53,371 | 74.7 | 18,099 | 25.3 | 1,209 | 8.1 | 5,336 | 38.0 | 9,685 | 47.4 | 1,869 | 8.5 |
| Rural | 8,741 | 6,597 | 75.5 | 2,144 | 24.5 | 132 | 7.9 | 621 | 35.2 | 1,200 | 45.5 | 191 | 7.1 |
| Unknown | 72 | 61 | 84.7 | 11 | 15.3 | <11 | 0.0 | <11 | 0.0 | <11 | 0.0 | <11 | 0.0 |
| **Census Tract Poverty Indicator** |  |  |  |  |  |  |  |  |  |  |  |  |  |
| <5% Poverty | 122,030 | 96,755 | 79.3 | 25,275 | 20.7 | 1,939 | 6.6 | 8,610 | 40.3 | 12,426 | 42.5 | 2,300 | 5.5 |
| 5-10% Poverty | 136,882 | 105,995 | 77.4 | 30,887 | 22.6 | 2,256 | 7.0 | 10,198 | 40.5 | 15,665 | 44.3 | 2,768 | 6.3 |
| 10-20% Poverty | 142,656 | 106,531 | 74.7 | 36,125 | 25.3 | 2,256 | 8.1 | 11,515 | 41.8 | 18,726 | 47.8 | 3,319 | 7.5 |
| >20% Poverty | 91,626 | 64,339 | 70.2 | 27,287 | 29.8 | 2,075 | 11.0 | 8,422 | 45.0 | 14,118 | 52.7 | 2,672 | 9.8 |
| Unknown | 41,157 | 30,054 | 73.0 | 11,103 | 27.0 | 745 | 8.6 | 3,068 | 39.2 | 6,355 | 46.7 | 935 | 8.5 |
| **SEER Registry Region** |  |  |  |  |  |  |  |  |  |  |  |  |  |
| East | 213,114 | 158,460 | 74.4 | 54,654 | 25.6 | 4,137 | 8.5 | 18,698 | 44.7 | 27,280 | 46.6 | 4,539 | 7.1 |
| Midwest | 47,032 | 34,784 | 74.0 | 12,248 | 26.0 | 887 | 8.7 | 3,888 | 40.9 | 6,428 | 49.5 | 1,045 | 7.2 |
| South | 105,183 | 79,258 | 75.4 | 25,925 | 24.6 | 1,906 | 8.8 | 7,237 | 37.7 | 14,428 | 45.7 | 2,354 | 7.2 |
| West | 169,022 | 131,172 | 77.6 | 37,850 | 22.4 | 2,650 | 6.5 | 11,990 | 39.8 | 19,154 | 46.6 | 4,056 | 7.1 |

**Supplementary Table 2.** Associations between patient sociodemographic, clinical and tumor characteristics and emergency department (ED) involvement among SEER-Medicare patients diagnosed with breast, colorectal, lung, and prostate cancers (2008-2017), excluding patients with imputed index dates

|  | **All Cancer Sites**  **(N=465,357)** | **Breast**  **(N=109,749)** | **Colorectal**  **(87,566)** | **Lung**  **(N=125,450)** | **Prostate**  **(N=142,592)** |
| --- | --- | --- | --- | --- | --- |
| **Sex^1^** |  |  |  |  |  |
| Male | Ref | N/A | Ref | Ref | - |
| Female | 1.01 (1.00-1.02) | - | 1.02 (1.00-1.03) | 1.01 (1.00-1.02) | N/A |
| **Age in Years^1^** |  |  |  |  |  |
| 66-69 years | Ref | Ref | Ref | Ref | Ref |
| 70-74 years | 1.07 (1.05-1.08) | 1.11 (1.04-1.19) | 1.07 (1.04-1.11) | 1.02 (1.00-1.04) | 1.16 (1.09-1.23) |
| 75-79 years | 1.19 (1.17-1.20) | 1.32 (1.24-1.41) | 1.22 (1.19-1.26) | 1.07 (1.05-1.09) | 1.42 (1.34-1.51) |
| 80-84 years | 1.37 (1.35-1.39) | 1.61 (1.51-1.73) | 1.42 (1.38-1.46) | 1.17 (1.15-1.19) | 1.97 (1.84-2.10) |
| >85 years | 1.68 (1.66-1.71) | 2.13 (2.00-2.27) | 1.76 (1.71-1.81) | 1.35 (1.32-1.37) | 2.65 (2.48-2.85) |
| **Race and Ethnicity^1^** |  |  |  |  |  |
| Non-Hispanic White (NHW) | Ref | Ref | Ref | Ref | Ref |
| Non-Hispanic Black (NHB) | 1.31 (1.29-1.33) | 1.54 (1.45-1.64) | 1.26 (1.22-1.29) | 1.24 (1.22-1.27) | 1.59 (1.51-1.68) |
| Hispanic | 1.17 (1.15-1.19) | 1.28 (1.18-1.39) | 1.11 (1.08-1.15) | 1.15 (1.12-1.18) | 1.41 (1.31-1.51) |
| Asian American | 1.00 (0.97-1.02) | 1.14 (1.02-1.28) | 1.01 (0.97-1.05) | 0.99 (0.96-1.02) | 1.03 (0.93-1.15) |
| Native Hawaiian or Other Pacific Islander (NHOPI) | 1.24 (1.15-1.34) | 1.62 (1.22-2.16) | 1.27 (1.09-1.48) | 1.12 (1.02-1.23) | 1.64 (1.22-2.20) |
| American Indian or Alaskan Native (AIAN) | 1.16 (1.08-1.24) | 1.33 (0.97-1.82) | 1.13 (1.01-1.26) | 1.10 (1.00-1.20) | 1.33 (1.02-1.73) |
| Mixed Race | 1.15 (0.96-1.38) | 1.27 (0.59-2.74) | 1.09 (0.75-1.58) | 1.18 (0.96-1.45) | 1.05 (0.49-2.24) |
| **Married or Domestic Partner^3^** |  |  |  |  |  |
| Yes | Ref | Ref | Ref | Ref | Ref |
| No | 1.23 (1.22-1.25) | 1.33 (1.26-1.40) | 1.22 (1.20-1.25) | 1.17 (1.15-1.19) | 1.49 (1.42-1.56) |
| Unknown | 1.08 (1.07-1.10) | 1.22 (1.15-1.30) | 1.08 (1.05-1.10) | 1.07 (1.05-1.09) | 1.09 (1.03-1.14) |
| **Year of Diagnosis^1^** |  |  |  |  |  |
| 2008-2009 | Ref | Ref | Ref | Ref | Ref |
| 2010-2011 | 1.06 (1.04-1.07) | 1.08 (1.01-1.14) | 1.07 (1.05-1.10) | 1.05 (1.03-1.07) | 1.07 (1.01-1.13) |
| 2012-2013 | 1.09 (1.07-1.10) | 1.05 (0.99-1.12) | 1.11 (1.09-1.14) | 1.07 (1.06-1.09) | 1.12 (1.05-1.19) |
| 2014-2015 | 1.11 (1.09-1.12) | 1.00 (0.94-1.06) | 1.16 (1.14-1.19) | 1.08 (1.07-1.10) | 1.13 (1.06-1.20) |
| 2016-2017 | 1.15 (1.13-1.16) | 1.05 (1.00-1.12) | 1.20 (1.17-1.23) | 1.13 (1.11-1.15) | 1.13 (1.06-1.20) |
| **SEER Tumor Stage^1^** |  |  |  |  |  |
| Localized | Ref | Ref | Ref | Ref | Ref |
| Regional | 1.52 (1.49-1.54) | 2.00 (1.90-2.10) | 1.30 (1.27-1.32) | 1.41 (1.38-1.45) | 1.12 (1.05-1.21) |
| Distant | 2.45 (2.41-2.48) | 9.54 (9.11-9.98) | 1.67 (1.63-1.70) | 2.15 (2.11-2.20) | 3.85 (3.68-4.04) |
| **Charlson Comorbidity Score (0-3+)^5^** |  |  |  |  |  |
| 0 | Ref | Ref | Ref | Ref | Ref |
| 1 | 1.05 (1.04-1.07) | 1.01 (0.96-1.07) | 1.06 (1.04-1.08) | 1.06 (1.04-1.07) | 0.94 (0.89-0.98) |
| 2 | 1.14 (1.13-1.16) | 1.13 (1.05-1.20) | 1.17 (1.14-1.20) | 1.12 (1.10-1.14) | 0.99 (0.93-1.06) |
| 3+ | 1.21 (1.20-1.23) | 1.19 (1.11-1.29) | 1.26 (1.23-1.29) | 1.20 (1.18-1.22) | 0.96 (0.90-1.03) |
| **County Geography^2^** |  |  |  |  |  |
| Metropolitan | Ref | Ref | Ref | Ref | Ref |
| Urban, non-metropolitan | 1.02 (1.00-1.03) | 1.08 (1.01-1.15) | 0.96 (0.94-0.99) | 1.00 (0.98-1.02) | 1.22 (1.15-1.29) |
| Rural | 0.91 (0.86-0.95) | 0.97 (0.79-1.20) | 0.83 (0.76-0.91) | 0.95 (0.90-1.01) | 0.96 (0.80-1.16) |
| **Census Tract Poverty Indicator^4^** |  |  |  |  |  |
| <5% Poverty | Ref | Ref | Ref | Ref | Ref |
| 5-10% Poverty | 1.04 (1.02-1.05) | 1.02 (0.97-1.08) | 1.02 (1.00-1.04) | 1.03 (1.01-1.05) | 1.11 (1.05-1.17) |
| 10-20% Poverty | 1.11 (1.09-1.12) | 1.12 (1.06-1.19) | 1.07 (1.04-1.09) | 1.10 (1.08-1.12) | 1.20 (1.14-1.27) |
| >20% Poverty | 1.18 (1.16-1.20) | 1.25 (1.17-1.33) | 1.10 (1.07-1.13) | 1.16 (1.13-1.18) | 1.40 (1.32-1.50) |
| **SEER Registry Region^1^** |  |  |  |  |  |
| East | 1.05 (1.04-1.06) | 1.15 (1.10-1.21) | 1.08 (1.06-1.10) | 1.02 (1.00-1.03) | 0.96 (0.92-1.00) |
| Midwest | 1.01 (0.99-1.02) | 1.12 (1.05-1.21) | 0.98 (0.95-1.00) | 1.00 (0.98-1.02) | 0.99 (0.92-1.06) |
| South | 0.97 (0.96-0.99) | 1.14 (1.07-1.22) | 0.93 (0.91-0.96) | 0.95 (0.94-0.97) | 0.98 (0.92-1.04) |
| West | Ref | Ref | Ref | Ref | Ref |

^1^Prevalence ratios estimated using generalized linear models with log link and Poisson distribution. Model 1 independent variables included age group, sex, race category, sex, SEER region, stage at diagnosis, year of diagnosis, count of outpatient, inpatient and emergency department visit days in the year prior to diagnosis (as 3 separate variables) and tumor site (for all cancers model only).

^2^Model 2 includes Model 1 variables and county geography.

^3^Model 3 includes Model 2 variables and marital status.

^4^Model 4 includes Model 3 variables and poverty category.

^5^Model 5 includes Model 4 variables and comorbidity index.
